# Impact of total gastrectomy plus perioperative PD-1 inhibitors on survival in locally advanced gastric cancer

**DOI:** 10.3389/fonc.2026.1771329

**Published:** 2026-02-03

**Authors:** Fan Zhang, Min Huang

**Affiliations:** Department of Oncology, The First People’s Hospital Affiliated to Yangtze University, Jingzhou, China

**Keywords:** locally advanced gastric cancer, PD-1 inhibitor, survival rate, total gastrectomy, XELOX regimen

## Abstract

**Background:**

The prognosis for locally advanced gastric cancer (LAGC) remains suboptimal with standard perioperative chemotherapy. Integrating PD-1 inhibitors into this regimen is a promising strategy requiring further validation.

**Objective:**

To evaluate the efficacy and safety of total gastrectomy plus perioperative PD-1 inhibitor (Sintilimab) in the management of locally advanced gastric cancer (LAGC).

**Methods:**

In this retrospective cohort study, 200 patients with LAGC undergoing total gastrectomy (January 2021 - November 2022) were categorized based on treatment received into an experimental group (perioperative sintilimab + XELOX, n=100) and a control group (perioperative XELOX alone, n=100).

**Results:**

The experimental group demonstrated the markedly higher rates of R0 resection rate (97%: 90%, *P* < 0.05), pCR (25%: 10%, *P* < 0.05), and MPR (35%: 20%, *P* < 0.05) as opposed to the control group. Survival analysis revealed significantly better outcomes in the experimental group: 3-year OS (48%: 30%, P = 0.009), median OS (34.7: 23.6 months, P = 0.004), 3-year DFS (40%: 25%, P = 0.001), and median DFS (30.4: 21.3 months, P = 0.007) Two groups showed no clinically relevant difference in the frequency of grade ≥3 therapy-related adverse events (56.0%: 51.0%, *P* > 0.05). Immune-related adverse outcomes in the experimental group were mainly grade 1–2 hypothyroidism and rash, which were relieved after symptomatic treatment.

**Conclusion:**

Total gastrectomy combined with perioperative sintilimab +XELOX regimen for LAGC significantly improves radical surgery, pathological response rate and long-term survival, with a manageable safety profile, offering a potentially effective treatment strategy.

## 1 Introduction

Gastric cancer is the fifth most common diagnosed type of cancer nationwide (1). Although the diagnosis rate of early gastric cancer has increased with the widespread adoption of endoscopic technology, a significant number of patients are already found to be at locally invasive stages at the moment of confirmation (2, 3). For the locally advanced gastric cancer (LAGC), the current standard treatment modality is a comprehensive treatment centered on surgery, supplemented by systemic perioperative treatment to eliminate potential micrometastases (4). Total gastrectomy is a commonly used surgical approach that directly affects the long-term outcome of the patient (5). Intraoperative systemic chemotherapy has evolved into the standardized therapy option for LAGC, significantly improving the survival outcomes of patients (6). However, even with the standard treatment, their long-term overall survival rates are still unsatisfactory, with median overall survival (OS) ranges at around 40% (7). Postoperative relapse and metastases remain the primary causes of treatment failure, highlighting the urgency of developing more effective treatment strategies.

In recent years, immune checkpoint inhibitors (PD-1) have made major advances in the therapy of multiple solid tumors (8). Reports show that neoadjuvant PD-1 inhibitor monotherapy can achieve a pathological complete response (pCR) of 9.1% in patients with resectable gastric cancer (9). Meanwhile, neoadjuvant therapy has been proven to have advantages such as shrinking the primary tumor and eliminating microscopic metastases (10). At present, several research studies are preliminarily reported the promising prospects of perioperative chemotherapy targeting the immune system in resectable gastric cancer (11, 12). However, the results of these studies are not yet mature, and survival data specifically for “total gastrectomy” is still relatively scarce. In addition, patients who undergo total gastrectomy often face more significant problems such as nutritional disorders and weight loss, and the complexity of perioperative management is much higher than that of patients who have undergone distal gastrectomy (13). Against this backdrop, we hypothesized that the addition of sintilimab to perioperative XELOX would improve pathological response and survival rates in LAGC patients undergoing total gastrectomy. This study aimed to evaluate the impact of total gastrectomy in combination with perioperative PD-1 inhibitor treatment on survival outcomes in LAGC patients through retrospective analysis, with the expectation of providing robust medical evidence for the clinical application of this treatment modality.

## 2 Materials and methods

### 2.1 General information

A review of 200 patients diagnosed with LAGC in the Department of Gastrointestinal Surgery at our hospital between January 2021 and November 2022. These patients were randomly distributed into a control group (n=100) and an experimental group (n=100).

Inclusion criteria: (1) Confirmed gastric adenocarcinoma by gastroscopy biopsy and pathological examination; (2) The assessment by abdominal enhanced CT, endoscopic ultrasound and other examinations was cT3-4aN+M0 stage, which met the diagnostic criteria for locally advanced gastric cancer (14); (3) Eastern Cooperative Oncology Group (ECOG) Physical condition score 0-1; (4) Indicators such as hepatic and renal function, complete blood count, and blood coagulation function are generally normal.

Exclusion criteria: (1) Distant metastasis (M1) detected by preoperative examination; (2) Previous gastric cancer-related chemoradiotherapy, immunotherapy, or targeted treatment; (3) Past history of other solid malignancies (excluding previously treated skin basal cell cancer or invasive cervical carcinoma); (4) Presence of severe autoimmune disease, infectious disease or organ failure; (5) Allergic to the study drug (sintilimab, oxaliplatin, capecitabine).This study has been approved by the Medical Ethics Committee of Jingzhou First People’s Hospital, with the approval number (2024)CDYFYYLK(09-013).

### 2.2 Treatment methods

Selection of PD-1 Inhibitor: The PD-1 inhibitor sintilimab was chosen for this study based on several considerations. Firstly, it has demonstrated efficacy and a favorable safety profile in Chinese patients with gastric cancer in prior clinical trials and recent real-world studies (15, 16). Secondly, sintilimab is commercially available and covered by national health insurance in China for advanced gastric cancer, significantly enhancing its accessibility and feasibility for use in a perioperative regimen within our clinical setting (17). In contrast, older (≥ 70 years) patients had a higher risk of all-grade and high-grade irAEs when receiving camrelizumab chemotherapy combination treatment in study (18).

Both groups underwent total gastrectomy plus D2 lymph node dissection. Open surgery was chosen and completed by the same group of experienced surgeons. The surgical procedure strictly followed the norms of radical gastrectomy for gastric cancer.

1. Control group: Received perioperative XELOX chemotherapy regimen (capecitabine + oxaliplatin). Two cycles of perioperative chemotherapy, four cycles of perioperative chemotherapy, with each cycle lasting 3 weeks. Specific regimen: Oxaliplatin 130 mg/m² (Hubei Bantian Pharmaceutical Co., Ltd, National Drug Approval No.H20143170, specification: 50 mg, Batch Number: YB20200201-011), intravenous drip, day 1; Capecitabine 1,000 mg/m^2^(Qilu Pharmaceutical Group Co., Ltd, National Drug Approval No. H20133361, specification: 0.5 g, Batch Number: QL20200601-A), oral administration, twice a day, days 1-14.
2. Experimental group: Treated with the perioperative sintilimab + XELOX regimen. The chemotherapy regimen was the same as that of the control group, combined with sintilimab 200 mg[Innovent Biologics (Suzhou) Co. Ltd, National Drug Approval Number: S20280016, Specification: 200 mg x 1 bottle], intravenous drip, once every 3 weeks, in conjunction with chemotherapy, 2 cycles before surgery, 4 cycles after surgery, for a total of 6 cycles.

### 2.3 Observation indicators

The primary endpoints of this study were 3-year disease-free survival (DFS) and 3-year OS. Secondary endpoints included R0 resection rate, pathological complete response (pCR), major pathological response (MPR), and treatment-related adverse events.

1. Surgery-related indicators: operation time, intraoperative blood loss, number of lymph node dissections, postoperative hospital stay, R0 resection rate (R0 resection is defined as no tumor residue in pathological examination of the surgical margin.
2. Pathological indicators: pCR, which is defined to mean the lack of surviving cancer elements in the original tumor and cleared regional lymph nodes; MPR, as determined by remaining viable tumor elements occupying ≤ 10% of the tumor site area; Tumor regression grade (TRG) is an indicator of the degree of tumor cell necrosis or regression after preoperative chemotherapy or chemoradiotherapy. It can be classified as follows: (1) TRG grade 0: no residual tumor, that is, complete pathological response; (2) TRG grade 1: almost no residual tumor, with only a few cancer cells surviving; (3) TRG grade 2: Obvious tumor regression, but still with a considerable number of cancer cells; (4) TRG grade 3: No obvious tumor regression.
3. Survival indicators: The primary observation indicators were 3-year DFS and 3-year OS. DFS is the duration from randomized assignment to disease relapse, metastasis, or any cause mortality; OS is the duration from randomized assignment to any cause mortality.
4. Treatment-related adverse reactions: Record and grade all therapy-related toxicities according to the CTCAE 5.0 criteria, with particular attention to immune-related adverse events (irAEs), like rash, thyroid dysfunction, immune pneumonia, colitis, hepatitis, etc., as well as chemotherapy-related hematological and non-hematological toxicities. The five levels of CTCAE 5.0 include mild (Grade 1), medium (Grade 2), serious (Grade 3), life-endangering (Grade 4), and death (Grade 5).

### 2.4 Statistical analysis

Data was analyzed using SPSS 21.0 software. Descriptive statistics for quantitative data were indicated in mean ± standard deviation (`x ± s), and the groups were assessed using the *t*-test for unrelated samples. Contingency data were reported as counts (%), with intergroup comparisons were performed via the *χ²* test or Fisher’s exact test. Kaplan-Meier survival curves were plotted, and intergroup survivability comparisons were assessed with the log-rank test. Multivariate logistic regression analysis was conducted to identify factors independently associated with 3-year mortality. A probability value of P < 0.05 considered statistically significant.

## 3 Results

### 3.1 Comparison of baseline data between the two groups

There were no statistically significant differences (*P* > 0.05) in baseline characteristics between the two groups, including age, gender, tumor location, tumor size, ECOG performance score, and presence of comorbidities, indicating that the groups were well-balanced and comparable at baseline, as detailed in **Table 1**.

**Table 1:** Comparison of baseline data between the two groups.

Indicators	Control group (n=100)	Experimental group (n=100)	*t/χ^2^*	*P*
Age (years, $\bar{\text{x}}$± s)	56.81 ± 8.50	57.32 ± 9.20	0.407	0.685
Gender (n,%)			0.082	0.775
Male	58 (58.00)	56 (56.00)		
Female	42 (42.00)	44 (44.00)		
Tumor location (n,%)			0.098	0.952
Upper stomach	38 (38.00)	36 (36.00)		
Middle stomach	45 (45.00)	47 (47.00)		
Lower stomach	17 (17.00)	17 (17.00)		
Tumor size (cm, $\bar{\text{x}}$± s)	5.23 ± 1.30	5.41 ± 1.50	0.911	0.363
Clinical stage (n, %)			0.092	0.955
cT3N1M0	42 (42.00)	44 (44.00)		
cT3N2M0	45 (45.00)	43 (43.00)		
cT4aN1-2M0	13 (13.00)	13 (13.00)		
ECOG Score (n, %)			0.020	0.887
0 points	55 (55.00)	54 (54.00)		
1 point	45 (45.00)	46 (46.00)		
Complications (n, %)			0.271	0.873
Hypertension	29 (29.00)	29 (29.00)		
Diabetes	25 (25.00)	28 (28.00)		
No	46 (46.00)	43 (43.00)		

### 3.2 Comparison of surgery-related indicators between the two groups

**Figure 1:**
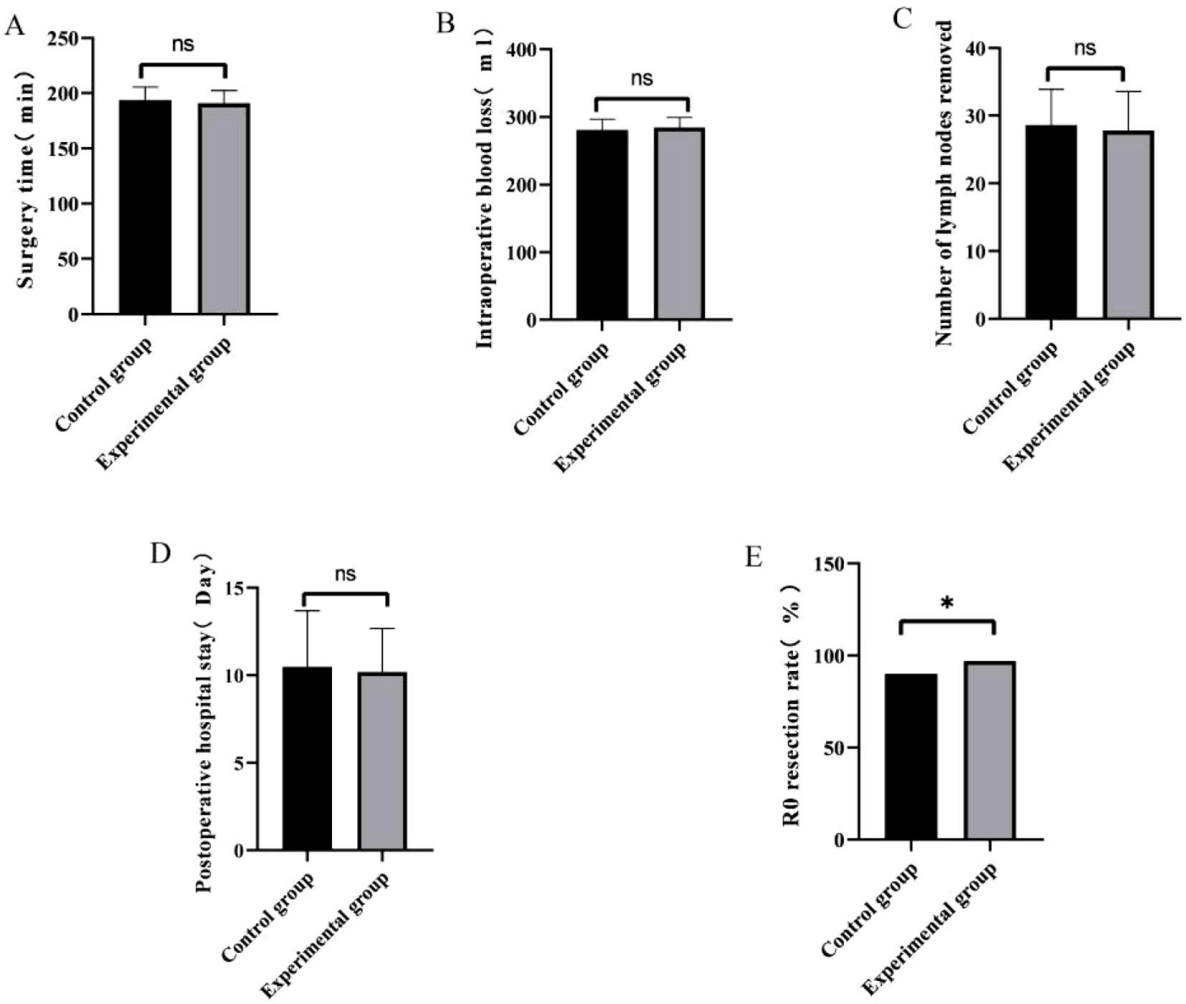
Chart comparing surgery-related indicators of the two groups of patients. **(A)** Comparison of the surgery time between the two groups; **(B)** Comparison of the intraoperative blood loss between the two groups; **(C)** Comparison of the number of lymph nodes removed between the two groups; **(D)** Comparison of the postoperative hospital stay between the two groups **(E)** Comparison of the R0 resection rate between the two groups. [n=100, ^*^P < 0.05, ns indicates no clinically significant correlation (*P* > 0.05)].

No statistically significant differences were observed between the two groups in terms of operative time, intraoperative blood loss, number of lymph nodes harvested, or postoperative hospital stay (all *P* > 0.05; **Table 2**, **Figures 1A-D**). However, the R0 resection rate was significantly higher in the experimental group (97%: 90%, *P* = 0.045; **Table 2**, **Figure 1E**).

**Table 2:** Comparison of surgery-related indicators between the two groups.

Group	Surgery time (min, $\bar{\chi }$ ± s)	Intraoperative blood loss (ml, $\bar{\chi }$ ± s)	Number of lymph nodes removed (, $\bar{\chi }$ ± s)	Postoperative hospital stay (Day, $\bar{\chi }$ ± s)	R0 resection rate (n, %)
Control group (n=100)	193.85 ± 11.58	284.65 ± 14.87	27.80 ± 5.80	10.50 ± 3.20	90 (90.00)
Experimental group (n=100)	190.98 ± 11.47	280.94 ± 15.96	28.60 ± 5.30	10.20 ± 2.50	97 (97.00)
*t/χ^2^*	1.761	1.701	1.019	0.739	4.031
*P*	0.080	0.091	0.310	0.461	0.045

### 3.3 Comparison of pathologically related indicators between the two groups

The pCR rate in the experimental group was 25%, and the MPR rate was 35%, markedly higher than the 10% and 20% rates in the control group, and these differences being statistically significant (*P* < 0.05). The proportion of tumor regression grade (TRG) 0–1 in the experimental group was 49%, markedly greater than 32% in the control group, and this difference was statistically significant (*P* < 0.05), as presented in **Table 3**.

**Table 3:** Comparison of pathologically related indicators between the two groups.

Group	PCR (n, %)	MPR (n, %)	TRG 0-1grade (n, %)
Control group (n=100)	10 (10.00)	20 (20.00)	32 (32.00)
Experimental group (n=100)	25 (25.00)	35 (35.00)	49 (49.00)
*χ^2^*	7.792	5.643	5.997
*P*	0.005	0.018	0.014

### 3.4 Sankey diagram of patient pathways

**Figure 2:**
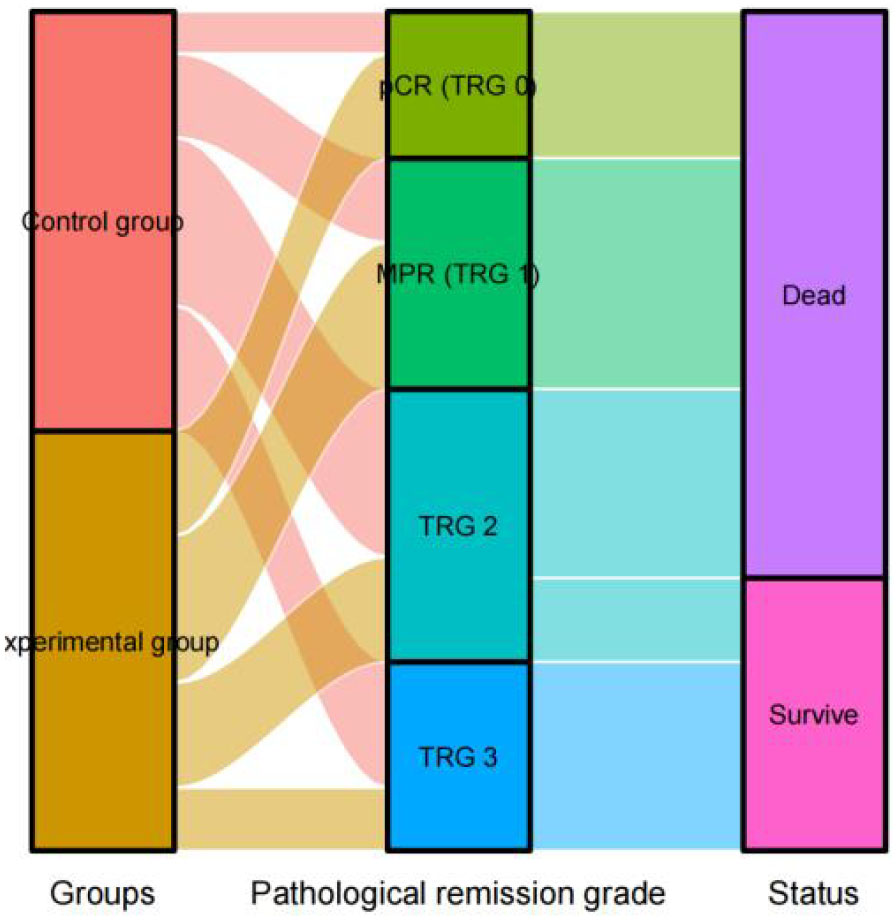
Sankey diagram showing the association between pathological response indicators and 3-year postoperative survival status.

To visually summarize the relationship between treatment assignment, pathological response, and 3-year survival outcome, a Sankey diagram was constructed (**Figure 2**). The diagram illustrates the flow of patients from the initial treatment group (Experimental or Control) through their achieved pathological response status (pCR/MPR or Non-pCR/MPR) to the final 3-year survival status (Alive or Deceased). This visualization confirms the trends observed in the statistical analyses: a greater proportion of patients in the experimental group progressed from treatment through favorable pathological responses to being alive at 3 years.

### 3.5 Comparison of survival indicators between the two groups of patients

**Figure 3:**
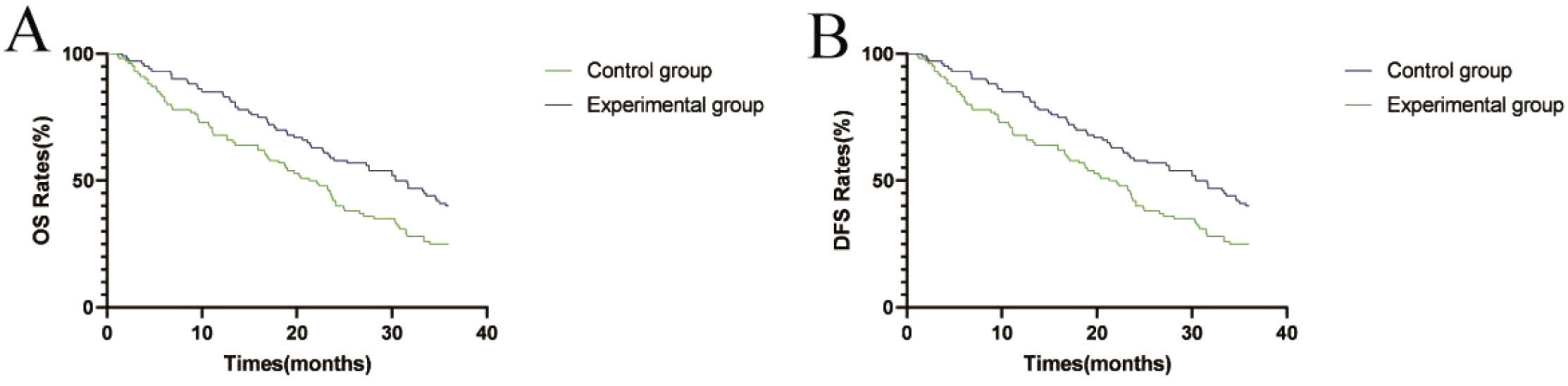
Survival curves of OS and DFS in the two groups. **(A)** OS survival curves of the two groups; **(B)** DFS survival curves of the two groups.

All patients completed the 3-year follow-up, with a 100% follow-up rate. The OS rates in the experimental group were 90% in the primary year, 65% in the next year, and 48% in the third year, which were significantly higher than 75%, 50%, and 30% in the control group, and the differences were statistically significant (*P* < 0.05); The median overall survival in the experimental group was 34.7 months and 23.6 months in the control group, indicating a statistically significant (*P* < 0.05); The DFS in the experimental group was 85% in the primary year, 58% in the next year, and 40% in the third year, which were significantly higher than 68%, 42%, and 25% in the control group (*P* < 0.05); The experimental group’s median overall survival was 30.4 months, while the control group’s was 21.3 months, indicating a clinically meaningful difference (*P* < 0.05). Specific data were shown in **Tables 4**, **5**; **Figure 3**.

**Table 4:** Comparison of OS between the two groups.

Group	1-year survival rate(%)	2-year survival rate(%)	3-year survival rate(%)	Median overall survival(months)
Control group(n=100)	75.00	50.00	30.00	23.6
Experimental group(n=100)	90.00	65.00	48.00	34.7
Statistics	*χ^2^* = 7.792	*χ^2^* = 4.604	*χ^2^* = 6.810	Log-rank=8.353
*P*	0.005	0.032	0.009	0.004

**Table 5:** Comparison of DFS between the two groups.

Group	1-year disease-free survival rate (%)	2-year disease-free survival rate (%)	3-year disease-free survival rate (%)	Median overall survival (months)
Control group (n=100)	68.00	42.00	25.00	21.30
Experimental group (n=100)	85.00	58.00	40.00	30.40
Statistics	*χ^2^* = 8.038	*χ^2^* = 5.120	*χ^2^* = 12.040	Log-rank=7.392
*P*	0.005	0.024	0.001	0.007

### 3.6 Comparison of adverse events between the two groups

#### 3.6.1 Treatment-related adverse events

Both groups of patients experienced varying degrees of therapy-related adverse events, mainly including gastrointestinal reactions, myelosuppression, and neurotoxicity during the therapy duration. The occurrence rate of grade ≥3 therapy-related adverse events was 56.0% in the experimental group and 51.0% in the control group, with this disparity not being clinically significant (*P* > 0.05), refer to **Table 6**.

**Table 6:** Comparison of treatment-related adverse events between the two groups.

Group	Gastrointestinal reactions (n, %)	Myelosuppression (n, %)	Neurotoxicity (n, %)	Hand-foot syndrome (n, %)	Abnormal liver function (n, %)	Grade ≥3 combined (n, %)
Control group (n=100)						51 (51.00)
Any level	73 (73.00)	68 (68.00)	48 (48.00)	33 (33.00)	18 (18.00)	
Grade ≥3	14 (14.00)	21 (21.00)	9 (9.00)	4 (4.00)	3 (3.00)	
Experimental group (n=100)						56 (56.00)
Any level	75 (75.00)	70 (70.00)	50 (50.00)	38 (38.00)	20 (20.00)	
Grade ≥3	13 (13.00)	25 (25.00)	11 (11.00)	4 (4.00)	3 (3.00)	
*χ^2^*	0.104	0.094	0.080	0.546	0.130	0.503
*P*	0.747	0.760	0.777	0.460	0.718	0.478

#### 3.6.2 Immune-related adverse events

Immune checkpoint inhibitor-specific irAEs were observed in the experimental group receiving sintilimab combination therapy, mainly manifested as hypothyroidism (20 cases), rash (13 cases), diarrhea (7 cases), while no reports were found in the control group. The overwhelming number of irAEs were grade 1–2 and were effectively controlled after discontinuation of administration, use of glucocorticoids and symptomatic supportive treatment. No treatment-related deaths occurred.

### 3.7 Multifactorial logistic regression analysis of whether patients died within 3 years postoperatively

Multifactorial logistic regression analysis was performed with whether patients died within 3 years postoperatively as the dependent variable, and R0 resection rate, PCR, MPR, and TRG grades 0–1 as the independent variables, and the assignments of each factor are shown in **Table 7**, and the results are shown in **Table 3**.The results showed that R0 resection rate, PCR, MPR, and TRG grade 0–1 were independent influencing factors for patients presenting with death (*P* < 0.05), as shown in **Table 8**.

**Table 7:** Assignment of values to factors.

Variable	Assignment
R0 resection rate	0=No; 1=Yes
PCR	0=No; 1=Yes
MPR	0=No; 1=Yes
TRG grades 0-1	0=TRG grades 2-3; 1=TRG grades 0-1
Status	0=Surviving; 1=Dead

**Table 8:** Multifactorial logistic regression analysis of whether patients died within 3 years postoperatively.

Variable	*B*	*S.E*	*Wald*	*P*	Exp(B)	95%confidence interval for EXP(B)	
						Lower limit	Upper limit
R0 resection rate	-1.025	0.351	8.452	0.004	0.359	0.182	0.708
PCR	-0.893	0.326	7.438	0.006	0.409	0.215	0.778
MPR	-0.761	0.309	6.037	0.014	0.467	0.248	0.878
TRG grades 0-1	-0.942	0.368	6.451	0.011	0.390	0.193	0.788
Constant	1.628	0.683	5.682	0.017	5.092		

## 4 Discussion

This retrospective study suggests that the addition of the PD-1 inhibitor sintilimab to perioperative XELOX chemotherapy in patients with LAGC undergoing total gastrectomy is associated with significantly improved rates of R0 resection, pathological response (pCR and MPR), and survival outcomes (OS and DFS), without a substantial increase in severe treatment-related toxicity.

Our findings align with the growing body of evidence supporting the integration of immunotherapy into perioperative strategies for gastroesophageal cancers. For instance, the KEYNOTE-585 phase III trial reported a higher pCR rate with pembrolizumab plus chemotherapy compared to chemotherapy alone (12.9%: 2.0%) (12). More recent updates from large phase III trials continue to support this approach. For example, the 3-year follow-up data from the CheckMate 649 trial confirmed sustained survival benefits with first-line nivolumab plus chemotherapy in advanced gastric/gastroesophageal junction adenocarcinoma (19). Furthermore, the RATIONALE-305 trial demonstrated a significant overall survival improvement with tislelizumab plus chemotherapy in the first-line setting for advanced gastric/gastroesophageal junction adenocarcinoma (20). Additionally, the KEYNOTE-859 trial further validated the survival benefit of combining PD-1 inhibition (pembrolizumab) with chemotherapy in a similar patient population (21). This marked improvement highlights the potential of immunotherapy to enhance tumor regression before surgery, possibly leading to better long-term outcomes. Meanwhile, PD-1 inhibitors have a good safety profile, with adverse events mainly manifested as rash, diarrhea, and thyroid dysfunction. Most of these immune-related side effects are mild to moderate and can be effectively controlled by corticosteroid-based symptomatic treatment. Importantly, the addition of PD-1 inhibitors to perioperative regimens does not appear to significantly increase the risk of surgery-related complications, such as delayed wound healing or postoperative infections. These findings provide support for integrating PD-1 inhibitors into standard perioperative strategies for resectable LAGC, offering a promising approach to improve both pathological and potentially survival outcomes without compromising surgical safety (22).

Based on this, this study analyzed the combined perioperative efficacy of sintilimab combined with XELOX regimen through a retrospective study. Results indicated that the experimental group yielded markedly better outcomes relative to the control group in regards to R0 resection rate, pCR and MPR, suggesting that the incorporation of PD-1 inhibitors could enhance T-cell recruitment and stimulation, thereby eliminating tumor cells (23). Additionally, a study demonstrated that PD-1 inhibitors administered in concert with systemic chemotherapy significantly increased pCR rates (22.4% vs. 4.8%) and T-stage downgrade rates when contrasted with standard chemotherapy monotherapy (24). In terms of survival outcomes, the 3-year overall survival rate and median overall survival of the experimental group in this study were significantly better than those of the control group, further confirming the long-term value of perioperative immunotherapy. This result echoes the findings of the CheckMate 577 trial in esophageal cancer, which confirmed that postoperative adjuvant nivolumab adjuvant nivolumab substantially prolonged the disease-free survival of patients undergoing neoadjuvant chemoradiotherapy, suggesting that the sustained effect of immunotherapy in the perioperative period may achieve long-term survival benefits by eliminating minimal residual lesions (25). Similarly, in late-stage gastric cancer, several Phase III studies have confirmed that PD-1 inhibitors as a single therapy or in addition with systemic chemotherapy can bring significant survival gains to patients with late-stage gastric cancer or junctional adenocarcinoma of the stomach and esophagus (19, 20, 26). The multivariate logistic regression analysis further strengthened these findings by identifying R0 resection, pCR, MPR, and TRG 0–1 grade as independent protective factors against 3-year mortality (all *P* < 0.05). This statistical evidence supports the mechanistic link between the improved pathological outcomes observed in the sintilimab group and the subsequent survival benefit. In regard to safety, the study found no clinically meaningful difference in the frequency of therapy-related adverse events of grade ≥ 3 between the two groups, suggesting that the induction of sintilimab did not substantially increase the burden of chemotherapy-related toxicity. The irAEs in the experimental group were mainly hypothyroidism, rash and diarrhea, and the vast majority were grade 1-2, which could be effectively controlled through standardized drug discontinuation and glucocorticoid treatment, and no therapy-related deaths were observed. This is in line with the known safety profile of PD-1 inhibitors. Its mechanism of action lies in suppressing the PD-1/PD-L1 axis to reinstate T-cell anti-tumor responses, but it may also cause the immune system to attack normal tissues (27).

### 4.1 Limitations and future directions

While our findings support the clinical integration of PD-1 inhibitors, future strategies must focus on personalization and overcoming resistance. Emerging approaches, such as novel delivery systems designed to modulate the tumor microenvironment, hold significant promise for enhancing immunotherapy efficacy. For instance, nanotechnology-based cytokine delivery platforms have been explored as a strategy to potentiate immune responses while mitigating systemic toxicity, as reviewed in the context of gastrointestinal cancers (28). Integrating such innovative strategies with current immunochemotherapy regimens represents a vital future direction for optimizing outcomes in LAGC.

Regarding postoperative follow-up, our institutional protocol relied on clinical assessment and CT imaging every 3–6 months for the first 3 years. To enhance the sensitivity of recurrence detection and enable earlier therapeutic intervention, future management strategies should integrate dynamic monitoring of minimal residual disease (MRD). Circulating tumor DNA (ctDNA) has emerged as a powerful biomarker for this purpose, with studies demonstrating its ability to detect MRD, predict recurrence risk, and provide a lead time over conventional imaging (29). Consequently, we propose that serial ctDNA testing be incorporated into follow-up protocols. A positive ctDNA result could guide timely clinical decisions, such as initiating or intensifying adjuvant therapy, potentially transforming postoperative management from a reactive to a proactive paradigm.

This study has several important limitations that should be considered when interpreting the results. First, the retrospective, non-randomized design introduces inherent risks of selection bias and unmeasured confounding. Although baseline characteristics were balanced (**Table 1**), residual confounding from unobserved factors cannot be excluded. Second, the sample size (n=200), while substantial for a single-center study, was not predetermined by a formal power calculation, which may limit the generalizability and statistical robustness of some findings, particularly for subgroup analyses. Third, the statistical analyses were predominantly univariate. We did not perform multivariate regression models (e.g., Cox proportional hazards models) or propensity score matching to adjust for potential confounding variables, which restricts causal inference. Furthermore, no correction for multiple comparisons was applied. Additionally, this study did not incorporate dynamic monitoring of minimal residual disease (MRD) using circulating tumor DNA (ctDNA) postoperatively. While MRD assessment is an emerging prognostic tool that could guide adjuvant therapy decisions, it was not part of our standard clinical protocol at the time. In future prospective trials, serial ctDNA analysis should be integrated to evaluate its utility in this treatment setting.

To address these limitations and build upon our findings, future research should prioritize prospective, randomized controlled trials with adequately powered sample sizes. Such studies should employ more sophisticated statistical plans, including pre-specified adjustment for key prognostic factors and correction for multiple testing. Additionally, as mentioned above, incorporating biomarker analyses (PD-L1, MSI, TMB) and MRD monitoring will be crucial to identify patient subsets most likely to benefit from perioperative immunotherapy and to understand the biological mechanisms underlying treatment response.

## 5 Conclusion

In conclusion, this study confirmed that for LAGC patients undergoing total gastrectomy, the addition of perioperative XELOX chemotherapy and sintilimab markedly improves the R0 resection rate, pathological response rate, and survival outcomes, with a manageable and controllable safety profile. These findings provide robust evidence-based support for the clinical adoption of this treatment regimen in specific high-risk patient populations. Moreover, the results offer valuable insights and a clear direction for further refining and optimizing perioperative therapeutic strategies in gastric cancer management.

## Data Availability

The original contributions presented in the study are included in the article/supplementary material. Further inquiries can be directed to the corresponding author.
